# Physical Activity and Mental Health Declined during the Time of the COVID-19 Pandemic: A Narrative Literature Review

**DOI:** 10.3390/ijerph191811230

**Published:** 2022-09-07

**Authors:** Christina Amo, Najla Almansour, Idethia S. Harvey

**Affiliations:** 1 Department of Education, Health, & Kinesiology, Texas A&M University, College Station, TX 77840, USA; 2 Department of Health Sciences, School of Health Professions, University of Missouri, 313 Clark Hall, Columbia, MO 65211, USA

**Keywords:** exercise, mental health, COVID-19

## Abstract

(1) Introduction: Mental health (MH) and physical activity (PA) share a bi-directional relationship, but most studies report MH as the outcome. With diminishing pandemic-related MH, this review examines the impact of diminished MH on PA. (2) Methods: This narrative literature review included 19 empirical studies published since the COVID-19 pandemic. Electronic databases such as MEDLINE, PsycINFO, and CINAHL were searched for English language articles in peer-reviewed journals using equivalent index terms: “anxiety”, “depression”, “stress”, “mental health”, “exercise”, “activity”, “COVID-19”, “coronavirus”, and “2019 pandemic”. The search reviewed 187 articles with double-rater reliability using Covidence. A total of 19 articles met the inclusion criteria. (3) Results: MH themes that impacted PA were depression and/or anxiety (*n* = 17), one of which identified inadequate coping and excessive pandemic stress (*n* = 2). In addition, women are more likely to suffer diminished MH and reduced PA throughout the pandemic. (4) Conclusion: Current research suggests that individuals with pre-pandemic MH episodes are correlated with more effective coping skills and fewer adverse effects from COVID-19 than expected. As we emerge from this pandemic, equipping all individuals, especially women, with positive coping strategies may accelerate a seamless return to PA.

## 1. Introduction

Physical activity (PA) is essential in maintaining health and living longer without chronic diseases by reducing the risk of certain chronic diseases and health outcomes through prescribed and preventative interventions [1,2,3,4]. The benefits of meeting PA recommendations include preventing and managing cardiovascular diseases [5,6,7], hypertension [6,8,9,10], cancer [10,11,12], stroke [12], diabetes [13,14], depression and anxiety [15,16], and improving overall well-being [13,17]. Since the 1970s, various organizations have invested tremendous efforts to advance PA advocacy in Healthy People 2020, now Healthy People 2030 [18]. Healthy People sets the United States public health agenda for a healthier society through physical activity [18].

The World Health Organization (WHO) and the Center for Disease Control and Prevention (CDC) recommend that early to midlife adults (i.e., aged 18–64 years) participate in 150–300 min of moderate-intensity (or 75 min of high-intensity) aerobic PA and two muscle-strengthening sessions at moderate intensity or greater per week [19]. Individuals who do not meet the minimum PA standards are 20% to 30% more at risk for mortality than individuals who meet the criteria [19].

Beyond the minimum PA requirements, it is recommended that individuals limit their sedentary time [20] (ST). Excessive ST is associated with all-cause mortality, cardiovascular disease, cancer, and type-2 diabetes [19]. The term “Sedentary Lifestyle Syndrome” (SLS), coined by Charansonney to describe long-term sedentary behavior, can be triggered by stress (i.e., a global pandemic) [21]. Prolonged “stay-at-home” orders increased sedentary behaviors, such as extended screen times and sitting (e.g., playing games, watching television, using mobile devices), and decreased PA. Prolonged ST hinders the body’s ability to cope with environmental challenges and increases the chances of acute health conditions (i.e., myocardial infarctions) [22]. Studies show that sedentary time has increased [23] since initiating COVID-19’s “stay-at-home” orders [23,24,25,26].

Before the COVID-19 pandemic, less than a quarter of American adults met the PA recommendations, according to the Centers for Disease Control and Prevention (CDC) website. SARS-CoV-2 (or COVID-19) was considered a global emergency by the WHO in January 2020, [27] requiring states to issue “stay-at-home” orders to prevent the spread of the virus [28]. These social distance mandates included mass shutdowns of popular PA resources (i.e., gyms, fitness centers, and parks) to slow the virus’s spread. This immediately and drastically reduced many Americans’ daily PA. Within just a few months of mandating these social distancing precautions, attempts to reverse diminished PA and increased ST were initiated. To encourage PA, many strategies and resources were mobilized. Work-place wellness programs provided virtual fitness classes and home exercise equipment [29]. Public health officials advised that outdoor activities (e.g., walking and running) [30] and community parks could allow individuals to exercise while maintaining physical distance [31]. Due to “stay-at-home” mandates, several gyms and fitness groups switched to physically distant or electronic options (i.e., e-lifestyles) for participants [32]. Individuals began to engage in PA using digital services [32]. Those with internet access had the resources to engage in e-lifestyles such as YouTube yoga, “live online” physical activity classes, and asynchronous walking/running programs. The American College of Sports Medicine also posted free “how-to” videos and other education for in-home PA programming [33]. Reduced PA is not as simple to treat as simply offering remote PA options. Despite all of these resources, after two years of chronic pandemic-related stress, PA has diminished even further [18].

SARS-CoV-2 is a novel virus, but traumatic global emergencies requiring social distancing are well documented. The COVID-19 pandemic has been compared to several past pandemics (e.g., the Spanish Flu of 1918, Severe Acute Respiratory Syndrome, or SARS, Ebola, and Middle East Respiratory Syndrome, or MERS) regarding mental health (MH) outcomes [34,35,36,37,38]. Lee and colleagues found that health care workers in contact with patients diagnosed with MERS reported high stress and anxiety levels resulting in post-traumatic stress disorders [36]. Sims anecdotally describe how the sudden outbreak of MERS resulted in increased anxiety within the community [37]. During the Spanish Flu, Americans suffered from fear, anxiety, depression, and other acute MH issues [38]. For the outbreaks of SARS and Ebola, research reveals the alarming severity of emotional distress [36]. As for MERS, studies further support that residual psychological trauma and overwhelming mental distress alter normal behavior [36,37]. These historical pandemics present evidence that individuals may experience MH consequences during traumatic situations [38]. Learning from history should make us more aware of the PA consequences of other MH events such as the COVID-19 pandemic. Because history affirms that stressful events are recurrent, understanding how diminished MH may impact PA is important to better prepare us in the future.

MH conditions continue to be a leading cause of disability domestically and internationally, and the COVID-19 pandemic may further increase its burden across the population [39]. Of all the MH conditions resulting from social distancing measures, depression, anxiety, and stress-related disorders are the most commonly reported. Like past pandemics, today’s pandemic mirrors historical consequences of isolation/quarantine, such as boredom, anger, and loneliness [38]. COVID-19’s social distancing measures have significantly affected the MH of people globally [40,41,42,43,44,45,46,47]. During COVID-19, the rate of depression increased five times [40,41], anxiety increased three times [42], PA decreased by 41% compared to pre-COVID-19 [43], and compliance with the standard PA recommendations decreased by over 80% [43]. It is not surprising as MH is a common comorbidity [46,47,48] for which PA is often recommended to treat or alleviate MH symptoms [46,47,48]. Those not able to isolate experience a heightened concern for common symptoms (such as cough and fever) that are now indicative of a COVID-19 infection, further exacerbating fear and anxiety for those actively limiting their own exposure [43]. Research has supported that these psychological factors, and others such as stress and social isolation, correlate with adverse health behaviors [44,45,46,47,48]. Psychological well-being promotes many healthy behaviors, while the lack thereof deters them [48].

As demonstrated, the complexity of stressful events can affect physical, social, mental, and general health [42]. Aptly, the COVID-19 pandemic has provided an opportunity to examine if diminished MH can actually be a precursor for decreased PA and potentially a contradiction for PA prescriptions [42]. Stanton et al. cross-sectionally surveyed 1492 adults in Australia and discovered that COVID-19’s social distancing measures negatively impacted PA (48.9%), sleep (40.7%), alcohol consumption (26.6%), and smoking (6.9%) since the onset of the COVID-19 pandemic [40]. They illustrated that these negative health behaviors in PA, sleep, smoking, and alcohol consumption were associated with increased depression, anxiety, and stress [40]. They further ascertained that long-lasting MH effects might arise from fear of infection, confusion, anger, post-traumatic stress symptoms, separation, frustration, boredom, lack of resources and information, financial loss, and stigma [40]. Likewise, other current literature identifies the most vulnerable psychological factors as isolation, fear, emotional stress, anxiety, and depression [38]. According to historically similar pandemics, any of these four acute MH illnesses may previse reduced PA [38]. The current pandemic allows us to analyze the MH and PA relationship in real-time compared to historical analysis from similar pandemics.

In summary, the bleak reality is that many people are neglecting their mental and physical well-being. This observation leads us to consider alternative barriers to PA during these events. The pandemic’s contribution to diminished MH promotes prolonged ST, decreased PA, and other adverse health behaviors [22,23,38]. In response to this recurrent co-occurrence and the known bi-directional relationship between PA and MH, we pose this research question: during excessively stressful events (i.e., the COVID-19 pandemic), does diminished MH serve as a barrier to PA? To answer this question, this review explores whether the pandemic’s MH impact is a significant barrier to PA. Therefore, the study aims to review existing literature for articles that present diminished MH as an indicator of decreased PA to evaluate this alternate direction. We hypothesize that countless studies measure both MH and PA, but only a few will infer that acutely diminished MH may negatively impact PA.

## 2. Materials and Methods

### 2.1. Design

A systematic review was conducted following the Preferred Reporting Items for Systematic Reviews and Meta-Analysis (PRISMA) checklist [49]. A comprehensive literature search with narrative methods was performed for this study to identify peer-reviewed articles addressing the role of mental health on physical activity during the COVID-19 pandemic.

### 2.2. Search Strategy

The following electronic databases were searched in March of 2022: MEDLINE, ScienceDirect (Elsevier), PsycINFO, Academic Search Complete (EBSCO), and the Cumulative Index of Nursing and Allied Health Literature (CINAHL), using keywords such as “anxiety”, “depression”, “stress”, “mental health”, “exercise”, “activity”, “walking”, “running”, “cycling”, “COVID-19”, “coronavirus”, “2019-NCOV”, “SARS-CoV-2”, “COV-19”, and “2019 pandemic”. Each database’s search terms (i.e., equivalent index terms and free-text words) ensured broad coverage of published studies in our review.

### 2.3. Eligibility Criteria

Each study meeting the eligibility criteria was included in the systematic review: articles published in peer-reviewed journals in English, published after 16 March 2020, included adult subjects 18 years and older, included both MH and PA factors, and used various research designs and methodologies. The exclusion criteria were articles published before 16 March 2020, articles without any connection to PA, articles that targeted pregnant women or actively COVID-positive patients as participants, and articles using PA as the predictor and MH as the outcome.

### 2.4. Study Selection

**Figure 1:**
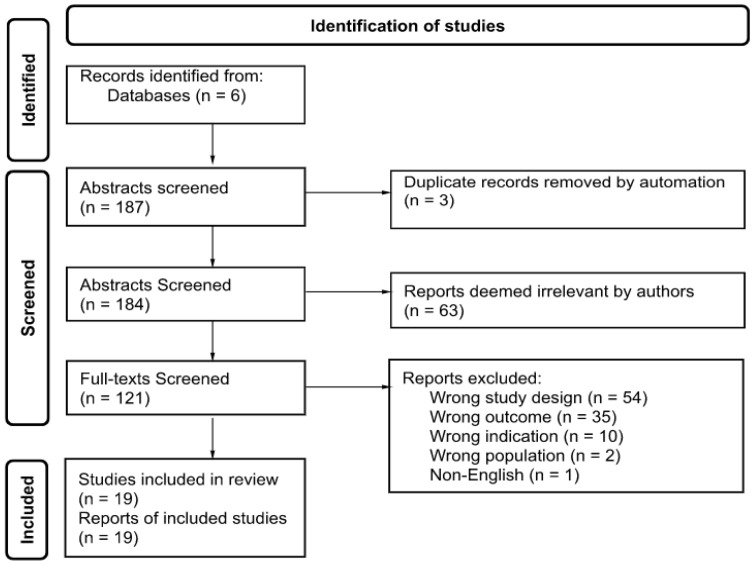
PRISMA flow diagram detailing the number of databases searched, abstracts and full-texts screened, and included.

The study selection consisted of three steps. First, each author independently screened all titles and abstracts (*n* = 187) of relevance for this systematic review [47]. In addition, the reference lists of all the included studies were scanned for relevant papers. Removal of three duplicate publications resulted in selecting 184 published papers. Second, the abstracts of all relevant articles were screened independently for eligibility by each author. Inter-rater reliability was 79%. The 63 articles deemed irrelevant were excluded. Third, the full papers of the included publications were obtained and screened for inclusion and exclusion criteria. The full text of 121 articles was reviewed. Furthermore, 102 articles were excluded, and 19 studies were selected to be analyzed. Figure 1 outlines the search process of the literature [48].

### 2.5. Data Extraction 

Each author independently selected data from the 19 studies using the data extraction tool. The authors met to compare the data and resolve inconsistencies by referring to the full-text article and thorough discussion. The following data were extracted: citation, country of study, the aim of the study, population demographics (e.g., age, gender), study designs, data source(s) (survey), and key observation(s) of the study (See Appendix A).

### 2.6. Quality Assessment

The assessment of each article’s quality was determined using the Joanna Briggs Institute (JBI) critical appraisal tools (See Appendix B) [50]. The criteria used JBI guidelines to evaluate whether each study is good quality and has minimal risk of bias. The study used a cross-sectional appraisal including eight criteria [51]. There are four answer choices in the JBI, namely “yes”, “no”, “unclear”, and “not applicable”. Conclusions were based on the results of the review. The more “yes” answers in the JBI critical appraisal column, the better and more valid the publication will be. The researchers independently evaluated the quality of each study, and disagreements were resolved by discussion within the review team. Identifying confounding factors and strategies to deal with confounding factors were not mentioned. This is of little concern for the current study. None of the studies were excluded based on their quality appraisal (see Appendix B).

### 2.7. Data Analysis

Due to the variability in the MH and PA measures, results could not be combined by meta-analysis. A narrative synthesis of the study was conducted. Tables and narrative summaries are used to present the study participant characteristics and the findings of the studies.

## 3. Results

### 3.1. Study Characteristics

All the studies were published in or after 2020. Out of the 19 studies, three of the studies were conducted in the United States [52,53,54], six of the studies were conducted in Europe [55,56,57,58,59,60], two of the studies were conducted in Asia [60,61], two of the studies were conducted in Australia [40,62], one of the studies were conducted in the Middle East [63], three studies were conducted in South America [64,65,66], one of the studies were conducted in Canada [67], and one of the studies was conducted in multinational region [68].

Out of 19 published studies, 18 employed survey data [40,43,52,53,54,55,56,61,62,63,64,65,67,68,69] and one employed observational data [60]. The study design for the published articles were longitudinal (*n* = 2) [56,60], retrospective (*n* = 1) [55], and cross-sectional (*n* = 16) [40,43,52,53,54,59,61,62,63,64,65,66,67,68,69]. Table A1 shows the selected characteristics of the 19 studies.

The sample sizes ranged from 58–41,923 individuals. Half of the studies (52.6%) had sample sizes below 500 [50,53,54,55,57,58,59,60,69,70]. One study focused on frail older adults (M = 82.4 years) [43], while six publications exclusively enrolled mid-to-late adults [40,52,56,60,62,63]. Most studies (84.2%) were primarily female participants [40,52,53,54,55,56,57,58,59,61,63,64,65,66,67,68]. Five publications did not report educational attainment [56,60,65,66,69] while several studies (47.4%) enrolled highly educated (i.e., college students or bachelor’s degree) participants [52,53,54,57,59,61,64,67,68].

### 3.2. Mental Health Assessment

Two studies used the 21-item Depression, Anxiety and Stress Scale (DASS-21) [40,62]. One study used the 16-item Quick Inventory Depressive Symptomatology tool [52]. One study used the Illness Attitude Scale [69]. One study used the Diagnostic and Statistical Manual of Mental Disorders, Version Four criteria (DSM-IV) [53]. Three studies used the Generalized Anxiety Disorder (GAD-7) [55,65,67]. One study used the Patient-Reported Outcomes Measurement Information System (PROMIS) [68]. One study used the Beck Anxiety Inventory tool [63] and one study used the Beck Depression Inventory tool [62]. Two studies used the Center for Epidemiological Studies-Depression Scale (CES-D) [56,61]. One study used Zung’s Self-reported Anxiety Scale (SAS) [57]. One study used the 14-item Hospital Anxiety Depression Scale [64]. Two studies used the Perceived Stress Scale (PSS) [54,60]. One study used the Yesavage Geriatric Depression Scale [43]. One study used a Mental Stress Indicator Score [59]. One study used a previous depression diagnosis [66].

### 3.3. Physical Activity Assessment

Six studies used a version of the IPAQ tool [54,55,62,63,65,68]. Six studies used self-reported PA frequencies [53,57,59,60,64,66]. Two studies used specialized items [52,56]. One study used four items for sport participation [69]. One study used the Yonsei Lifestyle Profile [61]. One study used the Active Australia Survey (AAS) [40]. One study used the PASB-Q tool [67]. One study used the Brief PA Assessment Tool (BPAAT) [43].

### 3.4. MH and PA Interaction 

Seventeen articles suggested that increased anxiety and depression reduced PA levels [40,43,52,53,54,55,56,60,61,62,63,64,65,66,67,68,69]. Stanton et al. found that community-dwelling adults reported increased anxiety, depression, and stress symptoms and decreased PA levels [40]. Coughenour et al. and Moriarty et al. found that college students reported higher levels of depression engaged in fewer minutes of PA [53,54]. Marashi et al. also reported that those whose MH symptoms increased significantly also reduced their PA during theCOVID-19 pandemic [67]. Those with more anxiety and depression reported a more significant decrease in PA level [67]. Stanton et al. found that females reported higher psychological distress scores than males [40]. Two studies reported that anxiety and PA were determined based on the participants’ behavior before the pandemic. Kaygisi reported that female participants who were less anxious during the pandemic were more likely to engage in sports before the pandemic [63]. However, Choi and Bum found that those who were anxious about being infected with COVID-19 were less likely to participate in sports activity during the COVID-19 pandemic [69].

Patients receiving medical care reported increased anxiety and depression reported decreased PA. Almandoz et al. reported bariatric patients who reported increased anxiety and depression reduced their PA levels by 47.9% [52]. Van Der Heide et al. reported that individuals diagnosed with Parkinson’s disease had an increase in poor MH outcomes and a decrease in PA due to worsened Parkinson’s disease symptoms [60].

One of these suggested that ineffective coping increases sedentary time [57]. Additionally, two studies supported that elevated stress levels reduced PA [54,59]. However, many studies also discussed other negative health behavior changes such as unhealthy eating habits, inadequate sleep, and other co-occurring behavior changes [40,54,55,56,59,61].

## 4. Discussion

Quarantine, isolation, and other social distancing measures are critical to reducing exposure to this novel virus. Unfortunately, the COVID-19 pandemic has had detrimental health consequences in both physical and MH. Some of the most concerning consequences discussed here include significant increases in emotional stress, depression, and/or anxiety paired with a continuous decline of PA. This mirrors the outcomes of historical pandemics that seem to repeat with extraordinary precision. This study provides evidence that acute MH illnesses such as anxiety, depression, and emotional stress can, in fact, hinder PA participation. Because diminished MH may impede PA, this study suggests that MH care should take precedence over PA reinstatement as we recover from the pandemic. These findings are unprecedented and greatly contribute to the existing literature by uniquely invoking the reverse directionality of the bi-directional relationship between MH and PA.

### 4.1. Research Implications

To our knowledge, there are no studies that identify the degree of or duration at which MH may impede PA. Longitudinal studies are needed to better understand the directionality of the bi-directional relationship between MH and PA. Until this is evaluated, we cannot accurately discern the significance of this impedance. Additionally, the degree of diminished MH that interferes with PA is important to accurately define and to guide PA recommendations. Currently, there is no existing acute MH assessment to indicate if physical abilities are impacted. Furthermore, evaluating populations with existing MH diagnoses would contribute toward even greater validity of this barrier. Lastly, it may be interesting to introduce MH practices such as meditation, relaxation, deep social connection, coping skills, and self-reflection into PA regimes to boost physical skills beyond current limits. In any manner, MH practices should be incorporated into all levels of healthy lifestyle interventions.

### 4.2. Study Limitations

Because this is a novel event with global attention, the demographics in these studies were reported in various ways, making generalizability difficult. Similarly, it should be noted that many of the included studies used predominantly female samples. Again, this makes generalizability less reliable. Because of the vastness in geographic locations and population cultural variations, MH and PA measurement tools varied. While most of the measurement tools were previously validated, they differed between each study. Due to the swiftness of related publications, it is possible that articles could have been incidentally omitted from this narrative review, especially those published in a language other than English and those that failed to explicitly compare the impact of diminished MH on PA. Lastly, and most notably, some cross-sectional studies assumed a potentially misleading causal relationship between MH and PA.

## 5. Conclusions

Obviously, more desirable pandemic-era PA behaviors would lessen post-pandemic physical health consequences. However, acute MH episodes may be a contraindication for concurrent PA participation in the current pandemic. Assessing one’s current stress levels may be a helpful tool for evaluating one’s PA readiness. Incorporating MH assessments into existing PA pre-screening protocols would help identify if an individual should prioritize MH practices before initiating a PA regimen. For better PA outcomes in future pandemics, preventative MH and coping practices appear to be necessary. To learn from previous shortcomings, it would be wise to exit this pandemic with MH strategies in mind for immediate and long-term utilization for all adults experiencing diminished MH, especially those most affected: women and older adults. Furthermore, providing standardized MH care as a long-term health prevention strategy may alleviate many other adverse health behaviors during future pandemics, including excessive substance use, inadequate sleeping patterns, poor nutrition, and sedentary and screen times. Even more grand, achieving optimal MH may induce the intended objectives of Healthy People 2030.

## Data Availability

Data sharing not applicable to this article. No new data were created or analyzed in this study.

## Appendix A. Characteristics of Included Studies

**Table A1:** Study descriptions including author, year, region, study design, sample demographics, measures, and key observations.

	Study Description			Study Sample				Assessment Tools		Outcomes
Author, Year	Country of the Study	Study Design	Aim(s) of the Study	Sample Size	Age M or Range	Gender %	Education	Mental Health	Physical Activity	Key Observation(s)
Almandoz, 2020 [52]	USA	Cross-sectional online survey	The study examined the relationship between COVID-19 psychosocial health implications among clinical obese adult patients and health professionals’ attitudes regarding COVID-19.	123	M = 51.2 (SD ± 13.0)	Female 87% (*n* = 107)	Bachelors or higher 56.1% (*n* = 69)	Quick Inventory of Depressive Symptomatology	Has the time you dedicated to exercise changed? Has the intensity of your exercise changed?	PA substantially decreased in duration and intensity (47.9%, 55.8%, respectively) among obese patients and they showed an increase in anxiety (72.8%) and depression (83.6%).
Choi, 2020 [69]	South Korea	Cross-sectional	The study analyzed the health concerns caused by COVID-19 and the intention of people to continue to participate in sports.	229	18–55+ years	Female 42.4% (*n* = 98)	N/A	Illness Attitude Scale	4 items for sport participation	Findings indicated that the individuals participated in group sports but were anxious about their health and intended to discontinue group sports activities. It was understood that the participants were slightly anxious about the possibility of infection due to increased spread of COVID-19 and that they would no longer participate in group sports.
Coughenour, 2021 [53]	USA	Cross-sectional online survey	The study examined PA minutes among college students before and after “stay-at-home” orders and determined if the changes were due to depression.	194	M = 25.11 (SD ± 7.84)	Female 72.2% (*n* = 140)	Current college students *n* = 100%	Diagnostic & Statistical Manual of Mental Disorders, Version Four (DSM-IV).	Patient Health Questionnaire (PHQ-9)	College students reported higher depression scores (*p* < 0.01) and reduced PA (*p* = 0.01) after “stay-at-home” orders were issued. There was a small but significant (*p* = 0.04) correlation between changes in total minutes of PA and depression scores. Seniors (*p* = 0.05) and Hispanic students (*p* = 0.03) were less likely to report worsening depression scores than first year and non-Hispanic white students. Asian students were significantly more likely to report decreased PA than non-Hispanic white students. This study suggests that COVID-19 and its consequences may contribute to reduced PA and greater depressive symptoms in college students and that sub-groups of college students have been affected differently.
Czenczek-Lewandowska, 2021 [55]	Poland	Retrospective, pre-post survey study	The study assessed whether and to what extent the COVID-19 pandemic has affected the health behaviors of young adults and assessed the level of generalized anxiety and its impact on health-related behaviors.	506	M = 24.67 (SD ± 4.23)	Female 70.2% (*n* = 355)	Bachelors or higher 46.0% (*n* = 233)	Generalized Anxiety Disorder (GAD-7) scale	IPAQ-Short Form	During the COVID-19 lockdown, generalized anxiety negatively impacted sedentary behaviors and sleep quality.
Ding, 2021 [68]	Brazil Bulgaria China India Ireland Malaysia North Macedonia Singapore Spain Turkey USA	Cross-sectional	The study investigated the associations of country-level COVID-19 risk, mental health symptoms, demographic factors with PA engagement, level of PA, and change in PA during the COVID-19 lockdown.	11,775	18–65+ years	Female 63.7% (*n* = 7498)	Bachelors or higher 62.9% (*n* = 7406)	Adult Patient Reported Outcomes Measurement Information System (PROMIS) Short Form v1.0-Anxiety 4a and PROMIS Short Form v1.0-Depression 4a	IPAQ-7 item	The study reported that higher depression symptom mean scores were associated with greater odds of being sufficiently inactive and decreased PA levels during the lockdown. Higher mean anxiety scores were associated with greater odds of decreased PA level during the lockdown.
Kaygısı, 2020 [63]	Northern Cyprus	Cross-sectional online survey	The study examined exercise habits before and during the pandemic among post-menopausal women’s PA levels and anxiety. The study examined the factors related to the PA levels among post-menopausal women who self-quarantined during the pandemic.	104	50–70 years	Female 100% (*n* = 104)	N/A	Beck Anxiety Inventory	IPAQ-Short Form-Turkish version	The results showed that the post-menopausal women who exercised before the pandemic had higher PA levels during the pandemic. Post-menopausal women with more grandchildren engaged in less PA and reported higher anxiety levels. The levels of anxiety and PA were negatively associated with each other.
Kekäläinen, 2021 [56]	Finland	Longitudinal surveys	The study examined pre- to in-pandemic changes in health behaviors and depressive symptoms and investigated the role of personality in these changes among middle-aged Finnish females.	358	51–59 years	Female 100% *n* = 358	N/A	Center for Epidemiological Studies- Depression Scale (CES-D)	The frequency, intensity, and duration of leisure-time PA and recorded as MET hours per day were collected among participants. Perceived change in PA was asked about in the in-pandemic-I and -II questionnaires with the question: “Have you changed your PA or exercise behavior during the COVID-19 pandemic?”	Females reported more depressive symptoms and unhealthier eating habits at the end of the emergency conditions compared to the pre-pandemic time. An increase in depressive symptoms was associated with changing to unhealthier eating habits. Higher extraversion was associated with a perceived decrease in alcohol consumption and with changing to healthier eating habits. Females with higher neuroticism reported changing to either healthier or unhealthier eating habits. In general, some females reported healthier lifestyle changes while other females reported the opposite. Personality traits help understand these individual differences inadaptation to the pandemic situation.
Lara, 2021 [57]	Spain	Cross-sectional	The study examined which coping strategies and styles were associated with anxiety levels and determined whether PA during the COVID-19 pandemic differed in coping strategies and styles.	200	18–74 years	Female 70% (*n* = 140)	Bachelors or higher 57.5% (*n* = 115)	Zung’s Self- Reported Anxiety Scale (SAS), Spanish version	PA levels were self-reported before and during the pandemic weekly.	The study found PA as a coping resource. The study found that the prevalence of a sedentary lifestyle was four times higher during the pandemic than in the pre-pandemic stage (21% pre-pandemic, 87% during the pandemic). The negative change in PA was due to the restrictions to prevent the spread of COVID-19. The factors for decreased PA were the lack of home-based programming.
Marashi, 2021 [67]	Canada	Cross-sectional online survey	The study aimed to examine the relationship between PA and sedentary behavior and how it impacts perceived barriers and motivators to PA during the COVID-19 pandemic.	1669	18–65+ years	Female 82.4% (*n* = 1218)	Bachelors or higher 80.7% (*n* = 1026)	Generalized Anxiety Disorder 7-item Scale (GAD-7) Patient Health Questionnaire (PHQ-9)	PA and Sedentary Behavior Questionnaire (PASB-Q) was adapted to report PA and sedentary behavior 6 months before and during the COVID-19 pandemic	Participants reported higher psychological stress and moderate levels of anxiety and depression during the pandemic. Participants with the highest reported mental health deterioration were the least likely to be active. Most participants were unmotivated to exercise because they were anxious. The findings highlight the paradox between mental health and PA. People who wanted to be active to improve their mental health but found it challenging to be active due to their poor mental health. Likewise, participants who were more depressed were less motivated to engage in PA.
Martinez, 2020 [64]	Brazil	Cross-sectional	The study aimed to assess the changes in PA levels among Brazilians after social distancing measures were placed during the COVID-19 epidemic. The study also described the participants’ levels of anxiety and depression during the pandemic.	1613	18–60+ years	Female 63.1% (*n* = 1017)	Bachelors or higher 74.3% (*n* = 1198)	Hospital Anxiety Depression Scale	PA frequency was asked before the “stay-at-home” mandate and their perceptions of the impact of COVID-19 on their PA and life routines. The participants were asked if they were professional athletes or if they had a home gym.	The study found that participants who are against the social distancing measures on PA and do not agree that COVID-19 is a significant public health concern tend to have a higher prevalence of depression symptoms. Anxiety symptoms were reported lower among the participants who reported a low impact of social distancing on PA and daily life. The findings highlight the profound impact of the COVID-19 pandemic on the population’s mental health and show a global demand for strategies to improve the coping and adaptation process in a situation of social isolation.
Moriarty, 2021 [54]	USA	Cross-sectional survey	The study examined the relationship between perceived stress and health behaviors among college students during the COVID-19 pandemic.	868	M = 21.3 (SD ± 3.8)	Female 74.2% (*n* = 408)	College students 100% (*n* = 550)	Perceived Stress Scale (PSS)	International Physical Activity Questionnaire – Short Form (IPAQ-SF)	The findings indicated that reduced sleep and exercise were associated with higher stress levels regardless of degree programs among college students.
Park, 2021 [61]	South Korea	Cross-sectional online survey	The study investigated the impact of the COVID-19 pandemic on lifestyle, mental health, and quality of life.	104	M = 32.07 (SD ± 7.64)	Female 72.12% (*n* = 75)	Bachelors or higher 92.31% (*n* = 96)	Center for Epidemiological Studies Depression Scale (CESD)	Yonsei Lifestyle Profile	The study found significant reductions in PA and activity participation and decreased frequency and time during the pandemic compared to earlier periods.
Perez, 2021 [58]	Spain	Cross-sectional phone interviews	The study examined the PA changes among community-dwelling, frail older adults enrolled in a running program who were not diagnosed with COVID-19.	98	M = 82.4 years (SD ± 6.1)	Female 66.3% (*n* = 65)	Bachelors or higher 14.4% (*n* = 14)	Yesavage Geriatric Depression Scale	Brief PA Assessment Tool (BPAAT)	Overall, depressive symptoms decreased the odds of maintaining sufficient PA. Moreover, pre-lockdown mental health, frailty, and social relationships were associated with PA levels during the lockdown.
Puccinelli, 2021 [65]	Brazil	Cross-sectional	The study examined the impact of social distancing on PA levels and the association between depression and anxiety levels by gender while controlling for objective PA levels, PA changes during the pandemic, adhesion to social distancing, family members, and age.	1853	M = 38.6 (SD ± 12.4)	Female 60% (*n* = 1110)	N/A	Patient Health Questionnaire-9 (PHQ-9) General Anxiety Disorder-7 (GAD-7)	IPAQ	The study found that PA levels during social distancing were significantly lower than before the “stay-at-home” mandate, with 30% of the participants reporting moderate or severe symptoms of depression and approximately 23.3% moderate or severe symptoms of anxiety. Depression and anxiety levels were significantly associated with lower levels of PA, low family monthly income, and age associated with higher levels of anxiety and depression.
Rossinot, 2020 [59]	France	Cross-sectional	The study aimed to understand people’s behavior and mental state changes during COVID-19 quarantine.	1705	24–65 years	Female 63.5% (n = 924)	Bachelors or higher 84.3% (*n* = 1376)	Mental Stress Indicator	Self-reported PA change	The study found that negative mental state changes were strongly associated with nutrition, sleep, PA, and alcohol consumption. Confinement impacted every behavior studied except for nutrition. Almost 50.6% of the participants reported increased depression, stress, and irritability since COVID-19.
Shalash, 2020 [62]	Australia	Cross-sectional phone interviews	The study examined the impact of the COVID-19 pandemic on mental health, PA, and quality of life (QoL) among psychological distress patients.	58	Parkinson’s disease Patients M = 55.6 years (SD ± 9.956) Control M = 55.55 years (SD ± 5.708)	Parkinson’s disease Female 23.7% (*n* = 9) Control Females 30% (*n* = 6)	Bachelors or higher 0.0% (*n* = 58)	Depression, Anxiety, and Stress Scale-21 (DASS-21) Beck Depression Inventory (BDI)	International Physical Activity Questionnaire (IPAQ)–Short Form	Parkinson’s disease patients reported a negative impact on their mental health, PA, health care, and interest in virtual visits. Parkinson’s patients reported worsened stress, depression, and anxiety compared to the control group. Parkinson’s patients also reported decreased PA since the pandemic.
Stanton, 2020 [40]	Australia	Cross-sectional online survey	The study examined the association between psychological distress and changes in selected health behaviors since the onset of COVID-19 and related social isolation measures in Australia.	1491	M = 50.5 years (SD ± 14.9)	Females 67.4% (*n* = 999)	Years of education M = 16.3 (SD ± 5.1)	Depression, Anxiety and Stress Scale (DASS 21)	Active Australia Survey (AAS)	Depression, anxiety, and stress were associated with adverse changes in health behavior. Participants who reported a decrease in PA were likelier to report higher depression, anxiety, and stress symptoms.
van der Heide, 2020 [60]	Netherlands	Single-center, longitudinal observational study with an observation period of two years.	The study hypothesized that the COVID-19 pandemic increased psychological distress and decreased PA among patients diagnosed with Parkinson’s disease.	498	Responders M = 62.8 years (SD ± 9.0) Non-Responders M = 63.3 years (SD ± 9.1)	Responders Females 38.5% (*n* = 138) Non-Responders Female 44.3% (*n* = 62)	N/A	Perceived Stress Scale (PSS), Perceived Anxiety Scale (PAS), Ruminative Response Scale (RRS).	Self-reported changes in PA and minutes/hours of (moderate) intensive exercise per week	The findings found that the COVID-19 pandemic worsened Parkinson’s disease symptoms by evoking psychological distress and reduced PA.
Werneck, 2020 [66]	Brazil	Cross-sectional	The study examined the association between diagnosed lifetime depression, changes in physical activity, TV viewing, consumption of fruits and vegetables, and the frequency of ultra-processed food consumption during the COVID-19 pandemic.	41, 923	18–60+ years	Without Depression Female 50.8% (*n* = 17,801) With Depression Female 68.2 (*n* = 4693)	Bachelors or higher 35.5% (*n* = 14,883)	Previous diagnosis of depression	The frequency and duration of leisure-time physical activities before and during the quarantine period and classified using the cut-off point of 150 min per week.	Pre-COVID, individuals diagnosed with depression were more likely to have a higher prevalence rate of physical inactivity. During COVID, the physical inactivity incidence rate did not differ among people diagnosed with depression compared with the general population.

## Appendix B. Quality Appraisal for Selected Studies

**Table A2:** JBI critical appraisal checklist.

	Were the Criteria for Inclusion in the Sample Clearly Defined?	Were the Study Subjects and the Setting Described in Detail?	Was the Exposure Measured in a Valid and Reliable Way?	Were Objective, Standard Criteria Used for Measurement of the Condition?	Were Confounding Factors Identified?	Were Strategies to Deal with Confounding Factors Stated?	Were the Outcomes Measured in a Valid and Reliable Way?	Was Appropriate statistical Analysis Used?
Almandoz, 2020 [52]	Yes	Yes	Yes	Yes	Yes	Yes	Yes	Yes
Choi, 2020 [69]	Yes	Yes	Yes	Yes	Yes	Yes	Yes	Yes
Coughenour, 2021 [53]	Yes	Yes	Yes	Yes	Yes	Yes	Yes	Yes
Czenczek-Lewandowska, 2021 [55]	Yes	Yes	Yes	Yes	Yes	Yes	Yes	Yes
Ding, 2021 [68]	Yes	Yes	Yes	Yes	Yes	Yes	Yes	Yes
Kaygısız, 2020 [63]	Yes	Yes	Yes	Yes	Yes	Yes	Yes	Yes
Kekäläinen, 2021 [56]	Yes	Yes	Yes	Yes	Unsure	Unsure	Yes	Yes
Lara, 2021 [57]	Yes	Yes	Yes	Yes	Yes	Yes	Yes	Yes
Marashi, 2021 [67]	Yes	Yes	Yes	Yes	Yes	Yes	Yes	Yes
Martinez, 2020 [64]	Yes	Yes	Yes	Yes	Yes	Yes	Yes	Yes
Moriarty, 2021 [54]	Yes	Yes	Yes	Yes	Yes	Yes	Yes	Yes
Park, 2021 [61]	Yes	Yes	Yes	Yes	Yes	Yes	Yes	Yes
Perez, 2021 [58]	Yes	Yes	Yes	Yes	Yes	Yes	Yes	Yes
Puccinelli, 2021 [65]	Yes	Yes	Yes	Yes	Yes	Yes	Yes	Yes
Rossinot, 2020 [59]	Yes	Yes	Yes	Yes	Yes	Yes	Yes	Yes
Shalash, 2020 [62]	Yes	Yes	Yes	Yes	Unsure	Unsure	Yes	Yes
Stanton, 2020 [40]	Yes	Yes	Yes	Yes	Yes	Yes	Yes	Yes
van der Heide, 2020 [60]	Yes	Yes	Yes	Yes	Yes	Yes	Yes	Yes
Werneck, 2020 [66]	Yes	Yes	Yes	Yes	Yes	Yes	Yes	Yes
